# Peace or War?

**Published:** 1891-10-03

**Authors:** 


### The Hospital

A Weekly Journal of

**Science, Medicine, Nursing, and Philanthropy.**

*For Hospitals, Asylums, Infirmaries, Dispensaries, Medical Practitioners, Students, Nurses, and the Charitable Public.*

Vol. XI.—No. 262.] [Oct. 3, 1891.

### Peace or War?

"Still in thy right hand carry gentle peace to silence envious tongues." Wolsey was a great man, and Shakespeare was a greater. They had both probably learned by experience that there was a certain homely and every-day value in peace and sweet reasonableness. To do women justice, it must be admitted that as a body they are of much gentler mould than men, and have less pleasure in wrangling and fighting. But at the present time women are in somewhat of a mood of revolt; and among hospital nurses efforts are being made to stir up what seems to us an irrational, unjustifiable, and unprofitable strife. The intention appears to be to set nurses against hospital managers, and to strike up an offensive and defensive alliance with the medical officers of the institutions for the purposes of the war.

There are two things to be considered in entering upon a quarrel. The first is to determine whether or not the cause be just, and the second, to count the cost, both of victory and of defeat. It has been said that there is only one thing more terrible than defeat in war, and that is victory. The assailed party in the threatened hospital strife is the managing body. Now, either the lay managers of voluntary hospitals deserve to be assailed, or they do not. The mere fact that they are managers cannot justify an attack upon them, except, of course, in the minds of those inconsequent anarchists who are always "agin the Government," simply because it is the Government. To that class doctors at any rate ought not to belong. If there is an order of mind which is habitually conservative it is the medical. As little, one would think, ought women to rush into hasty and ill considered strife, merely because they are invited to do so in a loud brazen voice.

Let us put the hospital manager into the witness-box, or into the dock, if that be preferred. What are his crimes? The first that strikes one is, that he sends an annual cheque to the hospital funds; and the second, that he sends an additional and special cheque of larger amount every now and again when the buildings fall into disrepair, or a new wing is required, or nurses' dormitories and common rooms are demanded, or when the doctors want new instruments and appliances, or the Matron and the Secretary have outrun the constable in the way of general expenses. It must be admitted that a man who sends an annual cheque to a hospital and holds himself in a state of preparedness to give other cheques as they may be needed, has a very great deal to answer for. It is equally obvious that the doctor who does not give his annual and occasional cheques, but who, on the contrary, acquires professional elevation and honour from his connection with the hospital, is the very person who ought to call the lay manager to account, and to insist upon a complete and satisfactory explanation of the annual and occasional cheques. We admit to the full the right of the doctor to assail the subscribing manager on this ground.

It is not quite so clear that the nurse has a grievance against the lay manager on account of his subscriptions, still less does it appear that the patient would be justified in joining the forces of revolt. The nurse, however, undoubtedly has her own peculiar complaints to make. The lay manager is her employer, and finds the resources for her payment. He has shown the unpardonable indiscretion of selecting her from other candidates who sought training or paid employment at the particular hospital to which he and his fellow-managers have appointed her. Let it be cheerfully admitted that a young woman, educated or otherwise, who is selected by the lay management, educated in the hospital, profitably employed afterwards, and paid by the managers to a considerable extent out of their own pockets, has a very serious accusation indeed against those lay managers. The young woman should think of it, and should take decided and courageous steps both to make known far and wide her grievances, and to have them promptly redressed.

It occurs to the profane mind of the present writer, himself a physician, that there are two very effective methods of dealing with the distressing and painful grievances both of hospital physicians and surgeons and of hospital nurses. The lay manager must be resolutely put down. It must be made penal by statute for him either to build hospitals or to subscribe to them. If he persists in giving his wicked money for charitable purposes it must be promptly thrown into the Thames, or in the case of Manchester and Edinburgh into the Irwell and the Firth of Forth. In places where there are no rivers to throw the horrid money into, it might be proper to consider the propriety of pouring it down the hospital drains. In order that the lay manager may be completely and for ever extinguished, the next step to be taken will be to pull all the hospitals down. "Pull down the nests and the rooks will fly away," as certain fanatical vandals said when they razed the fine old Scotch cathedrals to the ground. It is admitted that those Presbyterians who destroyed the venerable ecclesiastical edifices of their country had weighty reasons for their work of demolition. And who shall deny that the unhappy doctors and nurses, who are trampled into the hospital mire by the utterly unscrupulous and tyrannical lay manager, would have equal and still greater justice on their side if they insisted upon pulling down and entirely demolishing every voluntary and endowed hospital in the kingdom?

Some readers may think we are unnecessarily severe in compelling attention to the present hospital ferment from this particular point of view. It is not our purpose to be severe at all, but only to express the very emphatic conviction that the strife which has been excited in various quarters is seriously to be deplored, and that there never can be either prosperity or happiness at any hospital unless the lay managers, the doctors, and the nurses, live in "unity of spirit and in the bond of peace."

Wrangling in any department of public and private life is a mark of weakness of character and of unworthiness of disposition. Party feeling, in Church and State for example, is not kept up by the leaders and stronger minds of the parties, but by the brainless spouters and silly gossipers who have not sense enough to take a just view of the situation, or who have wretched little private ends to serve by the fomentation of party strife. Does any one for a moment suppose that Lord Salisbury and Mr. Gladstone, Mr. Balfour and Mr. John Morley, Mr. W. H. Smith and Lord Rosebery could not in twenty-four hours settle the Irish question if they were delivered from the blazing firebrands and miserable little thistle-prickles of party organisation? As it is in the large world, so it is in the smaller world of the hospitals. There are persons without brains, and persons with private ends to serve. It is they who cause earthquakes and burstings asunder of private friendships and honourable public bonds.

What has charity—charity in its widest sense—to say to the quarrels of philanthropists? If we would but cease our wranglings for a brief moment we should hear her quiet voice. It speaks in tones of dignity and calm authority. The doctor who gives of his skill to the patient; the nurse who watches over him and tenderly ministers to his wants; the lay manager whose money provides him with the ward, the bed on which he lies, the medicines and the food, whose kindly administration bring back life to his body and hope to his mind—all these—doctor, nurse, and lay manager—are one. They cannot be separated without infinite disaster. It is a grave public scandal to see them at variance and at war. They must cease their foolish bickerings. The wiser among them must lead the less wise in the direction of peace. The nurse has been the cause of their quarrels; the nurse must unite them again. Is not this one of the noblest of all the duties to which man or woman can be called—to make peace where before were rage and war?

